# Intratracheal Injections

**Published:** 1891-01-10

**Authors:** 


### INTRATRACHEAL INJECTIONS.

Dr. Botey, of Barcelona, one of the most deservedly eminent of Spanish physicians, gives in *El Siglo Medico* an account of his experience in the introduction of fluid medicaments within the trachea, practised in the first instance experimentally on rabbits, and on himself, and afterwards on a number of patients. With the rabbits he injected the fluid directly through the walls of the trachea, drop by drop from a Pravaz' syringe. On his own person he used a syringe with a long curved nozzle, capable of passing between the vocal cords into the cavity of the larynx, which had previously been rendered insensitive by cocaine. With rabbits one gramme of water caused no inconvenience. Two gave rise to temporary asphyxia, relieved by artificial respiration, but on the animal being killed the next day, nothing abnormal was seen. The effects were the same with three grammes, but four injected within five minutes caused fatal suffocation. A gramme daily for nine days caused no irritation, as was shown by a post-mortem examination. Half a gramme of Pot. Bichrom. in a 0·5 per cent. solution caused little inconvenience, and no post-mortem alterations, though the same passed into the stomach by an œsophagus tube set up acute inflammation. He himself suffered no discomfort from successive daily injections of 14, 25, 37, and 50 grammes of water, beyond a slight fall in the frequency of the pulse and respirations. In a patient suffering from laryngo-tracheal syphilis he injected first 12, then 15, and next on 17 consecutive days 25 grammes of a one per cent. solution of iodide of potassium with 1 centigramme of perchloride of mercury, each injection thus containing 25 centigr. of potassic iodide, and 2½ milligr. of the perchloride. The disease, which had hitherto resisted all treatments, yielded "as if by magic," the patient being completely cured in a month.

